# Pulse wave signal-driven machine learning for identifying left ventricular enlargement in heart failure patients

**DOI:** 10.1186/s12938-024-01257-5

**Published:** 2024-06-22

**Authors:** Dandan Wu, Ryohei Ono, Sirui Wang, Yoshio Kobayashi, Koichi Sughimoto, Hao Liu

**Affiliations:** 1 Graduate School of Science and Engineering, Chiba University, Chiba, Japan; 2 Department of Cardiovascular Medicine, Chiba University Graduate School of Medicine, Chiba, Japan

**Keywords:** Pulse wave, Left ventricular enlargement, Heart failure, Machine learning, Weighted random forest, Densely connected convolutional networks

## Abstract

**Background:**

Left ventricular enlargement (LVE) is a common manifestation of cardiac remodeling that is closely associated with cardiac dysfunction, heart failure (HF), and arrhythmias. This study aimed to propose a machine learning (ML)-based strategy to identify LVE in HF patients by means of pulse wave signals.

**Method:**

We constructed two high-quality pulse wave datasets comprising a non-LVE group and an LVE group based on the 264 HF patients. Fourier series calculations were employed to determine if significant frequency differences existed between the two datasets, thereby ensuring their validity. Then, the ML-based identification was undertaken by means of classification and regression models: a weighted random forest model was employed for binary classification of the datasets, and a densely connected convolutional network was utilized to directly estimate the left ventricular diastolic diameter index (LVDdI) through regression. Finally, the accuracy of the two models was validated by comparing their results with clinical measurements, using accuracy and the area under the receiver operating characteristic curve (AUC-ROC) to assess their capability for identifying LVE patients.

**Results:**

The classification model exhibited superior performance with an accuracy of 0.91 and an AUC-ROC of 0.93. The regression model achieved an accuracy of 0.88 and an AUC-ROC of 0.89, indicating that both models can quickly and accurately identify LVE in HF patients.

**Conclusion:**

The proposed ML methods are verified to achieve effective classification and regression with good performance for identifying LVE in HF patients based on pulse wave signals. This study thus demonstrates the feasibility and potential of the ML-based strategy for clinical practice while offering an effective and robust tool for diagnosing and intervening ventricular remodeling.

## Introduction

In recent years, heart failure (HF) has become an increasingly significant burden on public health [1–3], while ventricular structure remodeling has been recognized as a pivotal process in the development of cardiovascular diseases, particularly the progression of HF [4, 5]. The determinants affecting the left ventricular remodeling: (I) the intensity, longevity, and quickness of elevation in pressure burden; (II) the volume load; (III) factors like age, racial/ethnic background, and gender; (IV) accompanying conditions including coronary artery disease, diabetes, obesity, and valve-related heart issues; (V) the hormonal environment within the nervous system; (VI) changes in the extracellular matrix; and (VII) hereditary influences [6]. Left ventricular enlargement (LVE), as a common manifestation of ventricular remodeling, is closely associated with symptoms such as cardiac dysfunction, HF, and arrhythmias [7]. The increase in left ventricular size due to LVE exerts a severe impact on the clinical prognosis of patients with mild with or without severe HF [8]. Recent studies have identified LVE as a precursor to left ventricular dysfunction and clinical HF in asymptomatic individuals [9, 10]. LVE is recognized as a risk factor for both the occurrence and mortality rates associated with cardiovascular diseases (CVDs) [11], serving as a critical indicator of cardiac events, closely associated with deteriorating cardiac function and unfavorable prognosis [12].

**Fig. 1:**
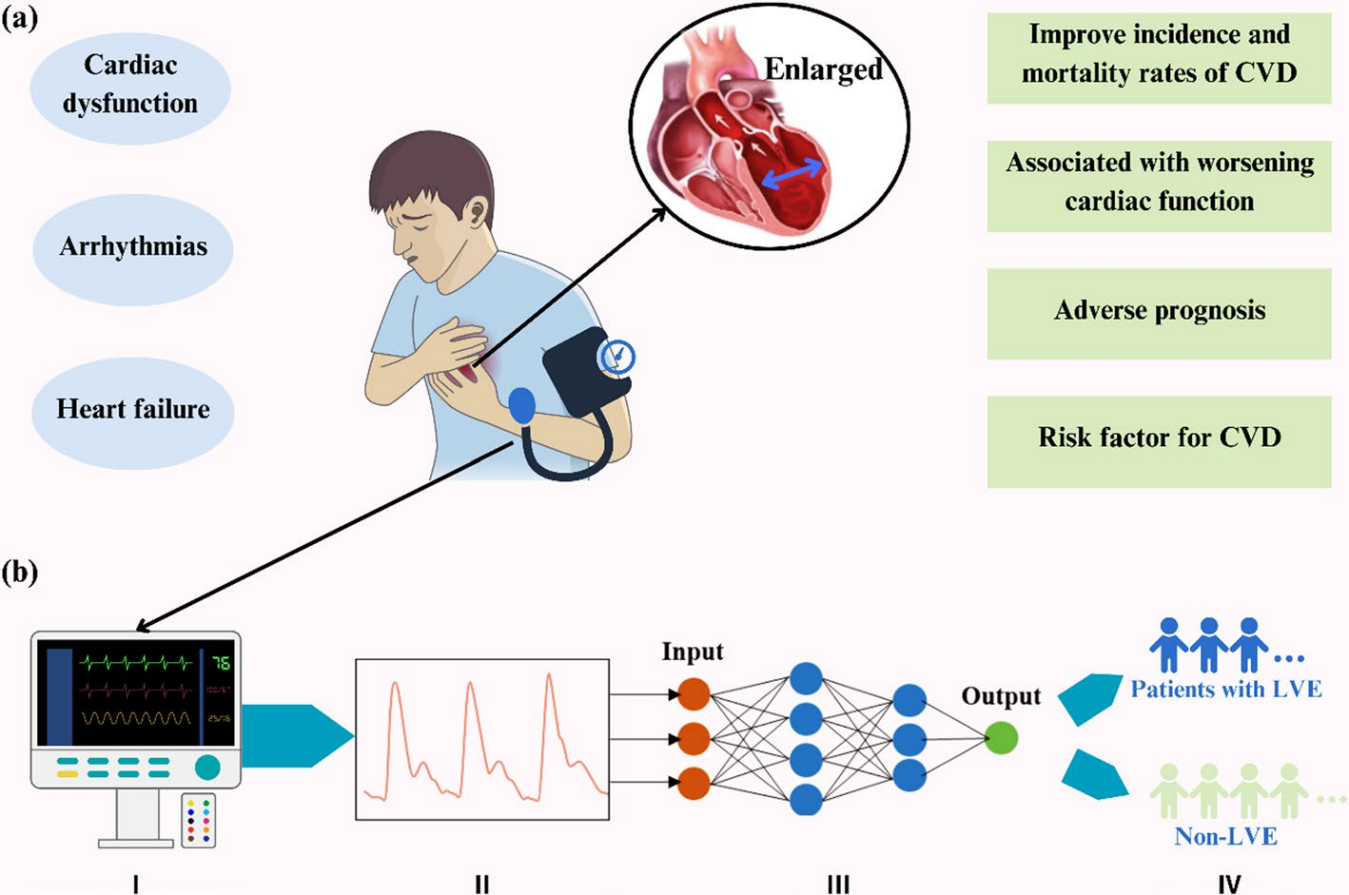
Illustration of **a** LVE and representative symptoms in CVDs, data-driven LVE detection, and **b** machine learning-based strategy for predicting LVE using pulse wave signals

LVE provides a crucial marker in cardiac pathology, and accurate evaluation of LVE facilitates the diagnosis and assessment of heart diseases and enables the prediction of cardiac events and prognosis, providing valuable guidance for medical treatments and management strategies. Thus, as illustrated in Fig. 1a, the monitoring and evaluation of LVE are particularly important from a clinical perspective. Detection of LVE primarily relies on the patient's medical history and imaging tests, with cardiac magnetic resonance (CMR) often being the most precise method due to its ability to accurately assess the heart's morphology, size, and position [13–15]. CMR is not routinely available due to its high cost, time-consuming, and magnetic field limitations for patients with metal implants [16, 17]. Consequently, transthoracic echocardiography (TTE), known for its noninvasive and real-time imaging, is widely regarded as an effective, patient-friendly method for evaluating and diagnosing cardiac functions [18]. However, the accessibility and convenience of TTE are limited due to the requirement for specialized equipment and skilled operators. This research leverages pulse waves as a key informational conduit within the cardiovascular system (CVS) to address these limitations and offer a noninvasive, cost-effective solution. Building on our previous study, we demonstrate that pulse waves can effectively infer CVS conditions and aid in diagnosing CVDs [19]. By exploring the use of pulse waveforms, this research identifies them as a promising tool for rapidly identifying and evaluating LVE (Fig. 1b).

The physiological signal of pulse waves has been widely utilized for health monitoring and CVD prediction [20–23]. Pulse waves contain valuable information on both physiological and pathological states of the human CVS while providing crucial physiological information associated with blood supply capacity and transportation efficiency [19, 24, 25]. Noninvasive measurements of pulse wave signals can now be easily implemented using various low-cost home electronic devices, providing helpful information for the low-cost and patient-friendly diagnosis of CVDs and relevant complications [26]. Recently, machine learning (ML) and deep learning (DL) methodologies have been employed for the analysis of pulse wave signals, demonstrating high potential and feasibility in terms of pulse wave pattern classification and cardiac function prediction [22, 27–30]. Wang et al. successfully classified 407 datasets of pulse waveforms into five patterns by developing a Bayesian network based on six pulse-waveform parameters of depth, width, length, frequency, rhythm, and intensity, achieving classification with a success rate of 84% [31]. Xu et al. classified 320 datasets of pulse waveforms into 16 patterns using a fuzzy neural network to extract the differences in pulse shape, width, position, and specific local parameters, enabling a classification success rate of 90% [32]. Li et al. proposed a CNN model to classify the pulse waveforms associated with five diseases of hypertension, atherosclerosis, hyperlipidemia, type 2 diabetes, and hypertension with concurrent atherosclerosis with a success rate of 95% [33]. More recently, in our previous study [19], Wang et al. established an optimized ML strategy that enables fast and accurate predictions of three cardiovascular functional parameters based on a pulse wave database of 412 patients, demonstrating the feasibility and potential of ML-driven, pulse wave-based predictions of cardiac function. In this study, we aim to propose and establish a pulse wave signal-driven ML-based strategy for identifying and evaluating LVE in HF patients and to provide a clinically effective, patient-friendly, low-cost, and noninvasive tool for early diagnosis and monitoring of CVDs.

## Results

### Fourier series calculation

**Fig. 2:**
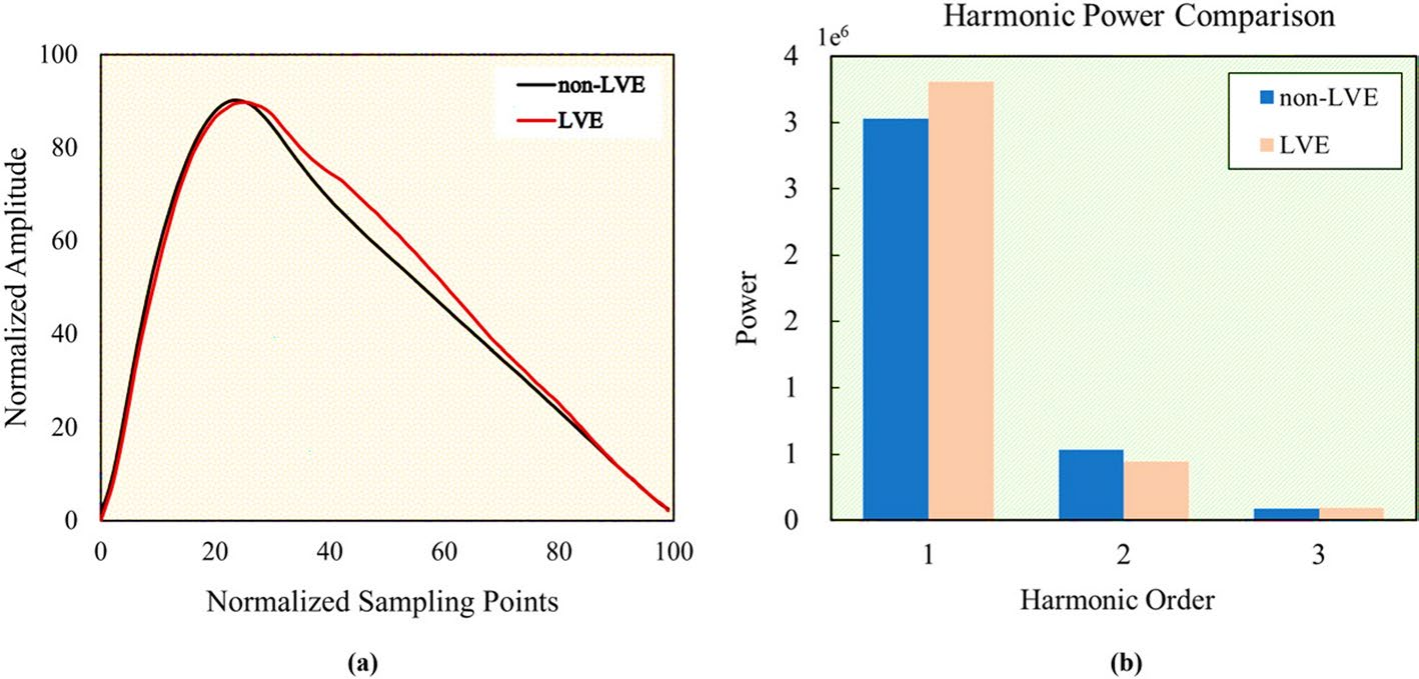
Comparison of representative pulse waveforms associated with LVE and non-LVE patients. **a** Normalized amplitudes vs. normalized sample points. **b** Harmonic powers vs. harmonic order

Through data screening and pre-processing, we successfully generated two high-quality pulse wave data sets specifically tailored to LVE and non-LVE patients. As shown in Fig. 2a, the representative waveforms of the two groups obtained based on K-means clustering show noticeable differences. Harmonic power decomposition (Fig. 2b) was performed using the 1st to 3rd order Fourier coefficients to examine how the harmonics contribute differently to the waveforms of the two groups. The pulse waveform in LVE is mainly dominated by the 1st order Fourier coefficient, but less by the 2nd order: the LVE group has a significantly higher power, but a slightly lower power than the non-LVE group, and there is rarely a discrepancy in the 3rd order. This implies that the LVE-induced irregular movement of the LV exerts an essential impact by the 1st order on dominating the feature of the pulse waves.

We further analyzed the harmonic frequency dependency of the two pulse wave datasets through independent sample t-tests, summarized in Table 1. In the frequency domain, we observed a significant difference between the 1st^−^ and 2nd-order harmonics (*p* < 0.05), while the influence of the 3rd-order harmonic was a margin (*p* > 0.05). This indicates that the datasets of the LVE and non-LVE groups are primarily affected by the 1st- and 2nd-order harmonics, while there is a notable frequency domain distinction between them. This provides further evidence of the validity associated with the dataset creation and an available guideline for data selection in subsequent classification and regression tasks, substantially enhancing the accuracy of pulse wave-driven identification of LVE.

**Table 1:** T-tests on the 1st-, 2nd-, and 3rd-order harmonics

Harmonic order	Non-LVE	LVE	*p*
1	3.11 ± 0.65	2.92 ± 0.74	0.048
2	0.61 ± 0.21	0.69 ± 0.35	0.027
3	0.13 ± 0.10	0.12 ± 0.09	0.363

Data are presented as the mean ± SD; *p* values were calculated using the independent samples t-test, where *p* < 0.05 represents significant differences

### Classification model

For a classification model performance test, the accuracy results of three models (WRF, SVM, and FCNN) are compared in Table 2. The WRF model shows the best classification performance with an accuracy of 91%: its specific classification metrics, as summarized in Table 3, for the LVE and non-LVE groups achieve an overall accuracy of 0.91, which is a remarkably high-accuracy classification, while the non-LVE group presents a slightly better performance (accuracy 0 = 0.93, recall H1 = 0.93, F1-score = 0.93) compared to the LVE group (accuracy 1 = 0.89, recall H2 = 0.89, F1-score = 0.89). This indicates that the current WRF classification model can achieve high prediction precisions with high recalls and F1 scores in identifying and predicting the most positive samples.

**Table 2:** Comparison of classification accuracy among the three methods

ML-models	Number	Accuracy
WRF	227	0.91
SVM	227	0.81
FCNN	227	0.77

**Table 3:** Classification performance of the WRF model

	Precision	Recall	F1-score	Support
Non-LVE	0.93	0.93	0.93	29
LVE	0.89	0.89	0.89	17
Accuracy		0.91		46

**Fig. 3:**
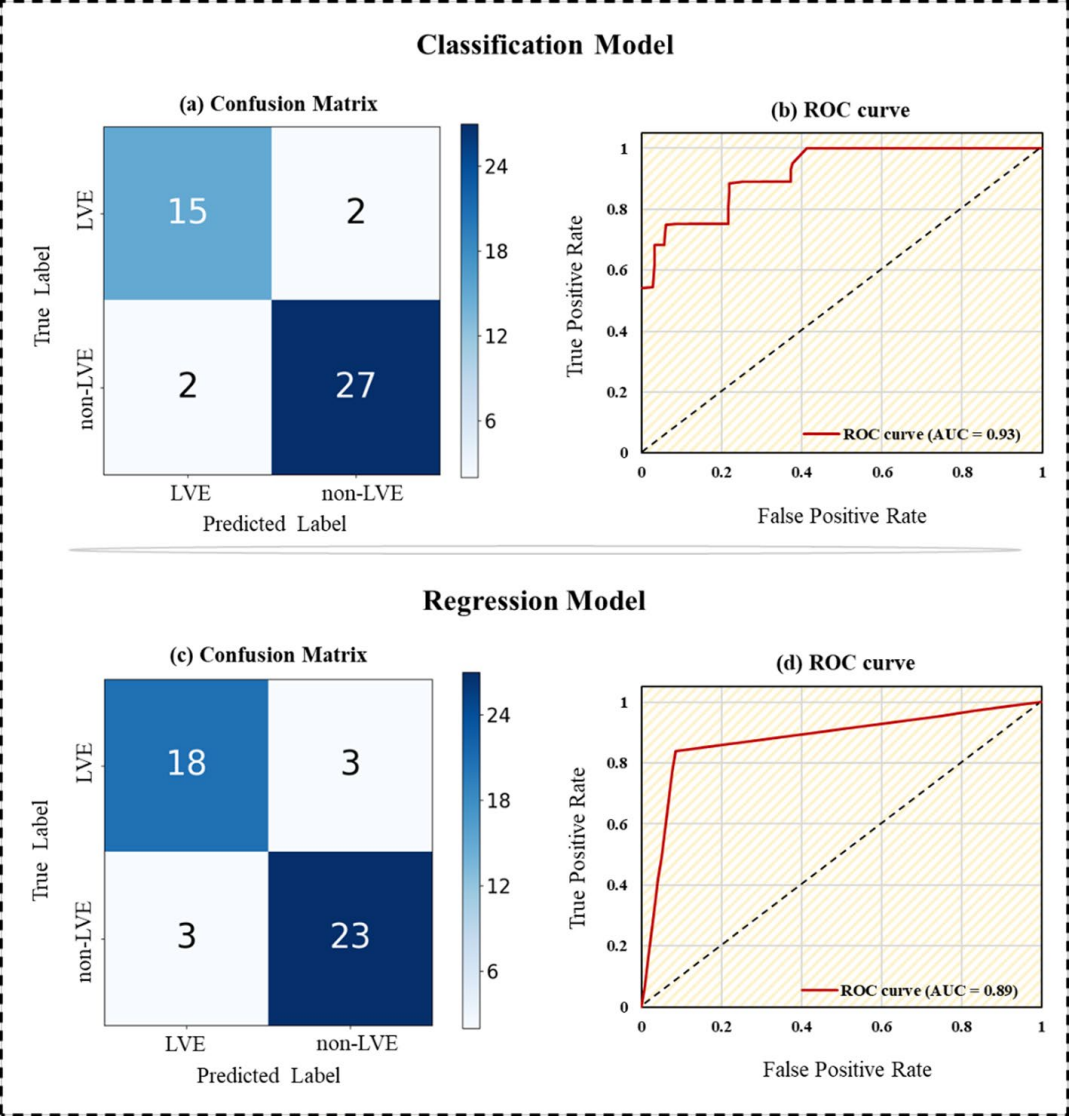
Confusion matrices and receiver operating characteristic (ROC) curves. **a** Confusion matrix of classification model. **b** Confusion matrix of regression model. **c** ROC curve of classification model, AUC = 0.93. **d** ROC curve of regression model, AUC = 0.89 (AUC: area under the curve)

The classification performance was further visualized using the confusion matrix, which, as shown in Fig. 3a, was employed to illustrate the classification outcomes of the LVE and non-LVE groups. While 2 out of 17 in the LVE group and 2 out of 29 in the non-LVE group were misclassified, the classification model overall demonstrated a capability to achieve the high-accuracy prediction of 93% for the non-LVE group and 88% for 5 LVE patients, even for the limited number of patients. To quantify the sensitivity and specificity of the classification model, we plotted the ROC curves in Fig. 3b, where a scalar metric of the AUC was employed at an AUC of 0.93 for the WRF model. The results indicate that the current classification model enables excellent differentiation between the LVE and non-LVE groups in up to 93% of cases compared to the perfect case of AUC = 1.

### Regression model

**Fig. 4:**
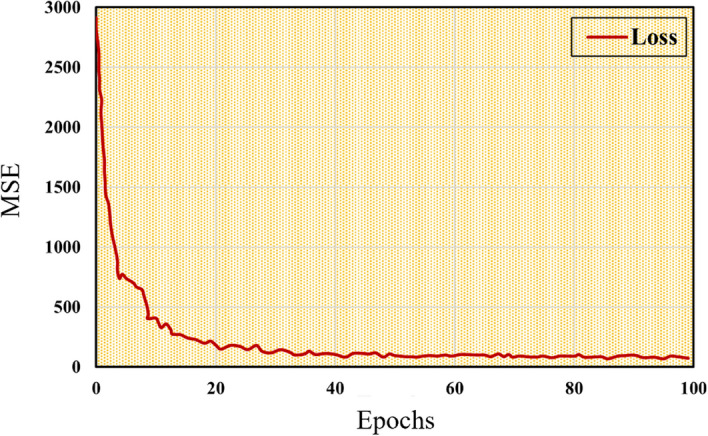
Learning the MSE curve of the DenseNet model

A Dense Net was utilized to predict the left ventricular diastolic diameter index (LVDdI) to optimize the ML network during the training process. The mean squared error (MSE), adopted as a loss function, displays a rapid and monotonic decline during the first several epochs (Fig. 4), converging quickly to a stable and minimum level after 100 training epochs. The relevant parameters and weights gained for the optimized model were then employed for the testing and machine learning-based predictions. As a result, the optimized regression model can achieve a prediction accuracy of 0.88.

**Fig. 5:**
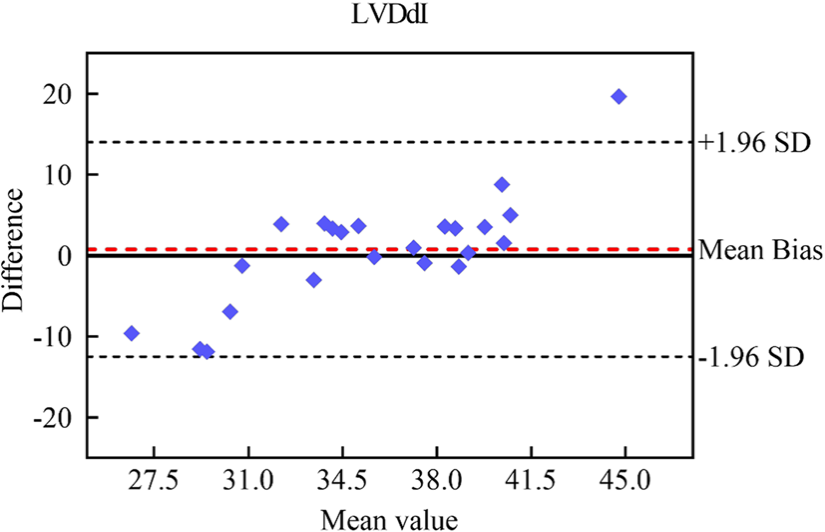
Comparison between Dense Net predictions and clinical measurements based on Bland–Altman analyses for LVDdI

To evaluate the consistency between the Dense Net predictions and clinical measurements, we applied the Bland‒Altman method to examine the mean values and differences, illustrated in Fig. 5, with an interval of 95% confidence. The Dense Net predictions are in good agreement with the clinical measurements, with most of the predicted LVDdI plots scattered within the 95% confidence interval. We presented the confusion matrix of the regression model based on the predicted LVDdI for a clearer comparison with the classification model, as shown in Fig. 3c. In this model, 3 cases from the LVE group and 3 cases from the non-LVE group were misclassified, which is slightly higher than the classification model. However, the regression model still demonstrated a high overall prediction accuracy of 88%. In addition, as shown in Fig. 3d, the ROC curve of the regression model, which estimates the LVDdI as a binary classification of the LVE and non-LVE groups, presents an AUC-ROC of 0.89, slightly lower than that of the classification model.

## Discussion

Left ventricular enlargement (LVE) plays a pivotal role in the clinical evaluation of HF. In this study, we demonstrate that employing pulse wave signals combined with advanced machine learning (ML) techniques can provide a promising avenue for quantitative identification with LVE and non-LVE in heart failure (HF) patients.

With two high-quality pulse wave datasets of 264 patients for the LVE and non-LVE groups, the ML-based prediction strategy was implemented using classification and regression models, which were validated through comparison with clinical measurements capable of achieving fast and accurate LVE identification in HF patients. Of the three established classification models, weighted random forest (WRF) model can achieve a remarkable differentiation between the LVE and non-LVE groups with a significantly high accuracy of 93%. In contrast, the dense net regression model enables an accuracy of 88% in directly predicting the left ventricular diastolic diameter index (LVDdI). The results demonstrate that the proposed methods and ML-driven methodology have high potential and feasibility to accomplish both LVE classification and LVE identification in HF patients based on pulse wave signals.

From a clinical perspective, the current pulse wave-driven, ML-based identification methodology can provide a noninvasive and low-cost tool for evaluating and diagnosing LVE in HF patients. As a noninvasive, real-time cardiac imaging technique to detect LVE, transthoracic echocardiography (TTE) is widely utilized. However, it has certain limitations regarding accessibility and convenience, which may lead to delayed medical treatments. This issue becomes even more crucial with expensive, high-sensitivity medical equipment such as nonenhanced multilayer spiral CT [34]. Recently, some attempts to utilize artificial intelligence methods have been conducted. Nam et al. [35] developed a way to detect left atrial enlargement (LAE) and LVE on chest X-rays using deep learning algorithms, but the results for LVE detection were all *p* > 0.05 compared to those for LAE, suggesting a need for inclusion of more images. The current ML-based methods demonstrate a patient-friendly and feasible tool for the first time to effectively differentiate LVE patients from the LVE and non-LVE groups using pulse wave signals. With the rapid advances in portable electronics and wearable devices such as smartphones and smartwatches, the noninvasive measurement of such physiological data has become more convenient and cost-effective. Pulse waves can thus be utilized for LVE detection, holding immense potential in assisting clinical management and treatment.

There are limitations in this study in terms of the relatively small sample size of data, the limited data scope, the singular data source, and a lack of pertinent clinical information. While we employed stringent methods in data selection and successfully created two high-quality datasets for classification and regression models, validation based on preliminary experiments remains open for further improvement; the data source from only one institution may not represent the diverse HF patient population. Additionally, the lack of healthy subjects and patients with other cardiovascular diseases may bring a potential bias, as it fails to encompass the entire HF demographic. To augment the generalizability of the ML models, there is an urgent need to expand the dataset by including a more diverse patient cohort. Further improvements in predictive accuracy and robust feasibility of the ML-based methodology to meet practical clinical applications may be accomplished by integrating multiple data sources, such as TTE and clinical biochemical markers, which will be our future endeavors. More efforts will improve the precision and reliability in training and testing with larger datasets, amalgamating relevant clinical information, and attempting patient classification based on LVE severity.

## Conclusion

In this study, the ML-based strategy successfully identified the patients with LVE in HF from those without LVE in HF. The proposed ML methods are verified to achieve effective classification and regression with excellent performance for identifying LVE in HF patients. This points to the potential and superiority of identifying patients with LVE based on pulse waves. Our study thus underscores the significance of the ML-based methodology for clinical practice, offering a robust tool for diagnosing and intervening in cardiac remodeling.

## Methods

**Fig. 6:**
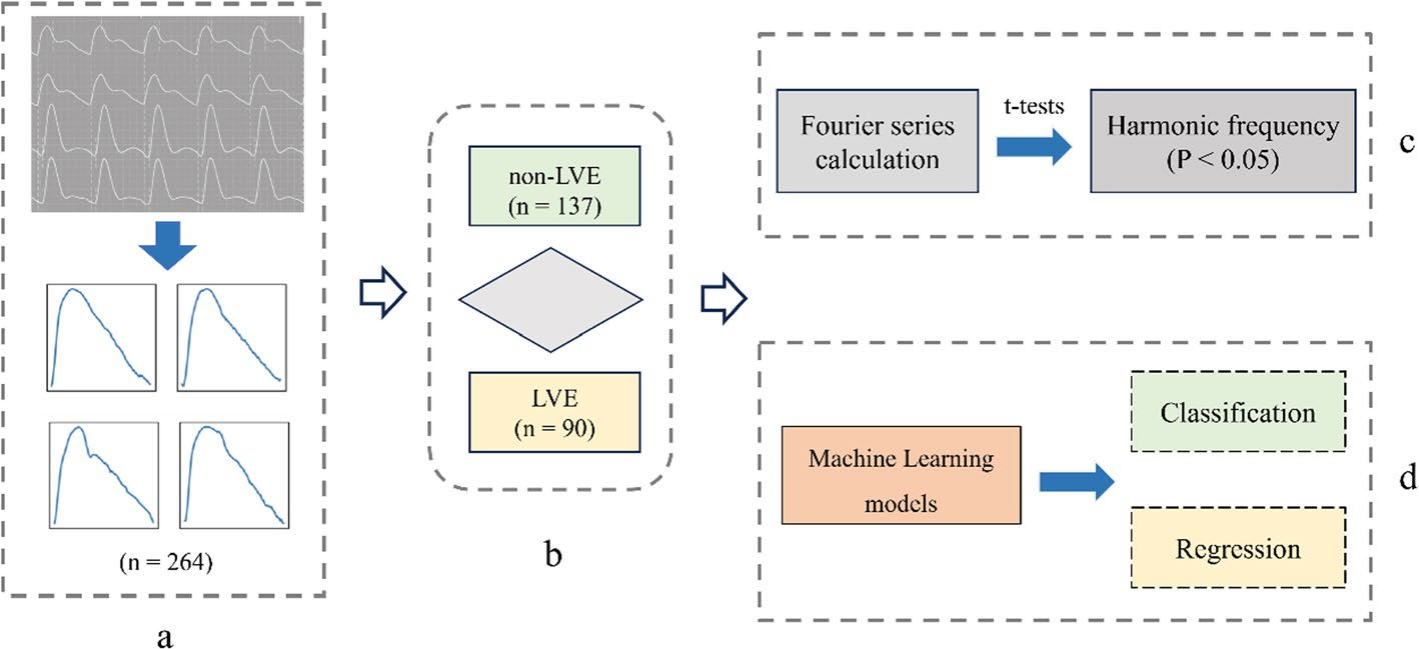
Framework of the machine learning process. **a** Data processing. **b** Data screening. **c** Fourier series calculation. **d** Machine learning models

Our machine learning framework consists of four parts: data processing, data screening, Fourier series calculation, and machine learning model analysis, as shown in Fig. 6:

### Ethics approval

This research received ethical approval from the Ethics Review Board of Chiba University Graduate School of Medicine in 2021 (Approval Number: M10089). The clinical data collection and analysis adhered to the applicable guidelines and regulations.

### Clinical data

All data were collected from 264 patients with HF, consisting of raw extremity pulse wave data and relevant clinical, physiological, and pathological information. HF was diagnosed based on the Framingham HF diagnostic criteria [36]. All patients were admitted to Chiba University Hospital between January 2019 and December 2022. After the acute HF condition stabilized, blood pressure/pulse wave detection equipment (Omron 203RPEIII) was used to measure and record the pulse waves and blood pressure. All patients underwent TTE (Vivid E9; GE Healthcare, Horten, Norway) within one week before or after the pulse wave tests. The measured parameters of relevant clinical information (e.g., age and body mass index) were also collected. None of the patients were confirmed to consume spicy food or alcoholic drinks during hospitalization.

### Pulse waves

Pulse wave signals were collected from the left upper arms of HF patients, followed by applying denoising and normalization techniques grounded in methodologies from previous studies [37, 38]. First, we utilized wavelet transform decomposition to remove noise from the signals [39]. Then, to prevent distortion of the pulse wave signals, we set the number of sampling points for each pulse wave cycle to 100, considering the Nyquist theorem and the actual sampling frequency [32, 40]. Since our study focused on variations in the pulse wave model, we normalized the pulse wave amplitude within each cycle to a range of 0 to 100.

### Datasets

Rigorous data screening was undertaken to ensure data quality. The 264 patients with HF were confirmed to satisfy the following screening criteria: (1) pulse wave data were collected from the left upper arms of the patients; (2) for each patient, five or more valid pulse wave cycles were recorded; and (3) LV size, including LV diastolic diameter (LVDd) and LV systolic diameter (LVSd), was measured by transthoracic echocardiography. Our clinical data were used to evaluate the LVE of each patient based on the guidelines provided by the American Society of Echocardiography (ASE) [36]. LVE was defined as LVDd with an index, namely, LVDdI > 36 mm/m2 for males and > 37 mm/m2 for females [12].

**Fig. 7:**
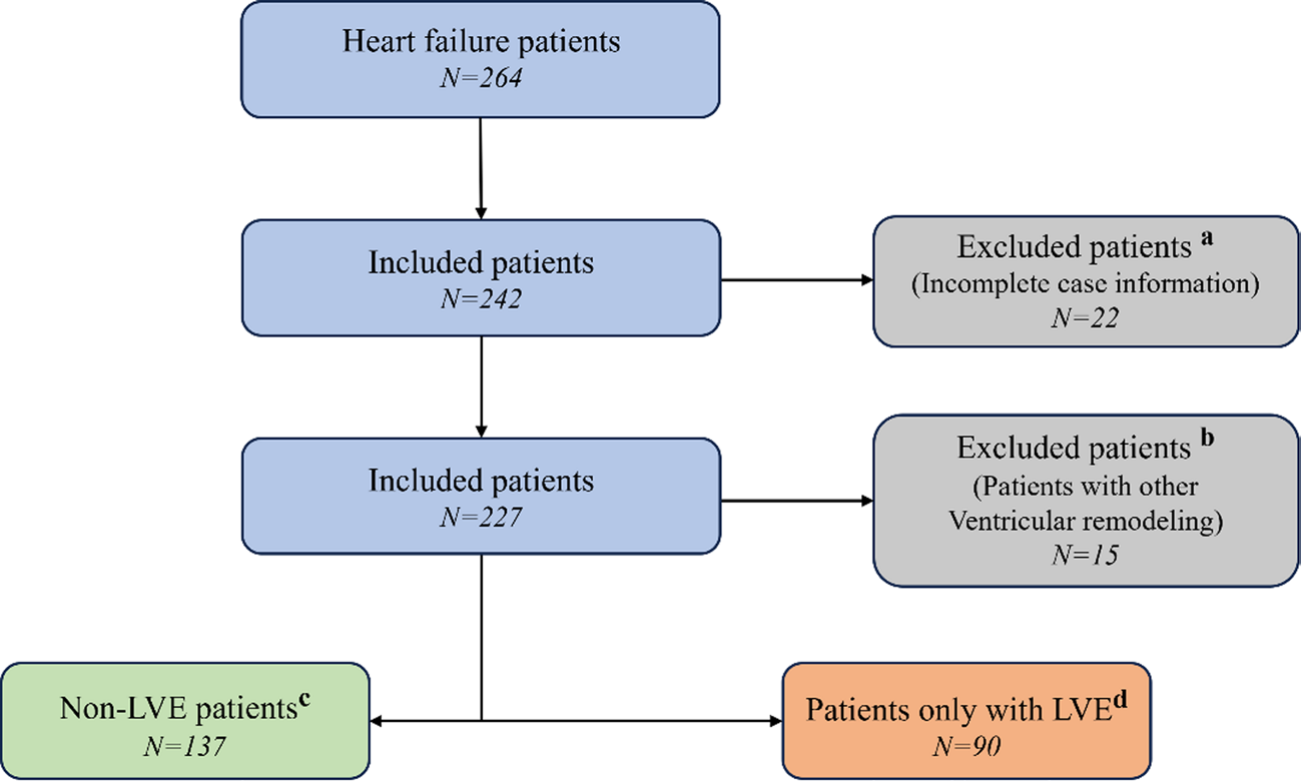
Flowchart of patient screening under screening criteria a, b, c, and d: (1) exclusion of 22 subjects with incomplete information^a^, (2) exclusion of 97 patients with other cardiac remodeling^b^, (3) creation of a non-LVE patient group of 137 Subjects^c^, and (4) creation of an LVE patient group of 90 Subjects^d^

The clinical data for 264 patients underwent classification, as depicted in Fig. 7. This dataset excluded 37 patients because of incomplete records. Among the remaining 227 qualified patients, 137 were identified as non-LVE patients, while 90 were diagnosed with LVE. The K-means clustering algorithm derived characteristic waveforms from these two datasets. These waveforms were then decomposed into 3rd-order Fourier series to explore the variances between the datasets, detailed as follows:

1 $$F\left( t \right) = \,A_{0} + A_{1} \cos \left( {\omega t} \right) + B_{1} \sin \left( {\omega t} \right) + A_{2} \cos \left( {2\omega t} \right) + B_{2} \sin \left( {2\omega t} \right) + A_{3} \cos \left( {3\omega t} \right) + B_{3} \sin \left( {3\omega t} \right),$$

where *A*_*0*_ is the period-average value, *A*_*1*_-*A*_*3*_ and *B*_*1*_-*B*_*3*_ are the 1st-order to 3rd-order Fourier series coefficients, respectively, *t* is time, and $\omega$ is the angular frequency. Independent sample t-tests were then conducted to statistically analyze the difference between the patient datasets with LVE and non-LVE.

### Machine learning models

Two ML models were developed and validated for classification and regression tasks. The classification model is a binary classifier that analyzes the pulse waveforms to examine whether the HF patients suffered from LVE; the regression model is employed to estimate the LVDdI based on the pulse waveforms.

#### Classification

In this study, our objective was to address the classification problem between patients with LVE and non-LVE. We constructed a pulse wave dataset using data from 227 HF patients. The dataset was divided into two subsets, with approximately 80% allocated to the training dataset and the remaining 20% to the testing dataset. To select an appropriate ML model, we conducted numerous preliminary experiments to test and compare the predictive performance among different models, such as the weighted random forest (WRF) model, support vector machine (SVM) model, and fully convolutional neural network (FCNN) method. Due to the imbalance in the data from the two groups of patients, we employed the WRF method, where different weights were assigned to the two groups during the training process. We utilized the class weight parameter of the random forest classifier from the scikit-learn library to adjust the weights based on the class distribution, aiming to improve the performance of the minority class. We extensively investigated various weight settings and substantially determined weight parameters of 1 and 4 for non-LVE and LVE patients, respectively. To mitigate the risk of overfitting, we used fivefold cross-validation and recorded the average scores to evaluate the model's performance more accurately.

#### Regression

The densely connected convolutional network (Dense Net) was employed for regression prediction of LVDdI, a recently established, innovative network architecture capable of excelling in efficient feature extraction and regression prediction tasks [41]. In the context of our limited dataset, the dense net is verified, enabling the effective employment of data features, and achieving high performance while mitigating overfitting. The relationship between the input and output of the (*n* + 1)th layer (feature map) associated with the dense block module is given as:

2 $$Output^{n + 1} = \,feature \,map = G^{n} \left( {Output^{1} , Output^{2} , \ldots ,Output^{n} } \right),$$

G denotes multiple operations, including the rectified linear unit (ReLU), batch normalization, and convolution.

**Fig. 8:**
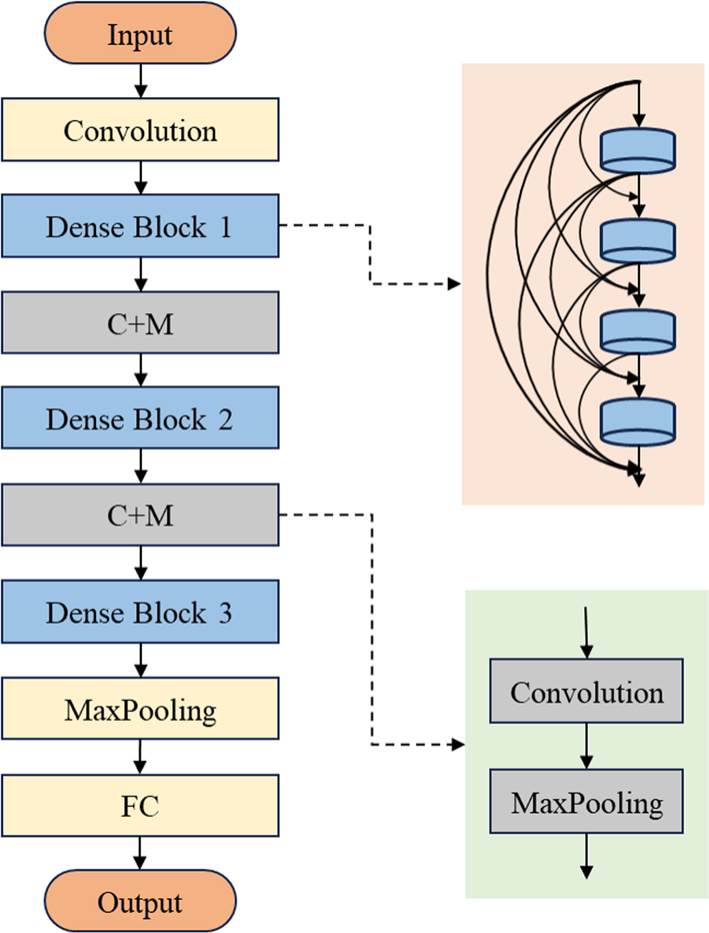
Schematics of densely connected convolutional networks (Dense Nets) for regression modeling

The specific network architecture, as shown in Fig. 8, consists of a dense net with two hidden layers: the 1st and 2nd hidden layers, which comprised 128 neurons and 32 neurons, respectively. The output layer has a single neuron dedicated to regression prediction. The ReLU is used as the activation function in the two hidden layers. Additionally, dropout layers are inserted between the 1st and 2nd hidden layers to mitigate overfitting.

The model was trained using the Adam optimizer under the following conditions: learning rate = 0.001, *ε* = 0.001, *ρ*_*1*_ = 0.9, *ρ*_*2*_ = 0.999, and *δ* = 1E − 8 [42] while using the MSE as the loss function. The ML networks were trained with TensorFlow (v2.0.0rc, Python 3.7) on an NVIDIA Quadro K4000 GPU, encompassing 100 epochs, with each batch having 128 samples. The model performance was evaluated for validation based on 10% of the training dataset.

### Performance evaluation

The performance of the weighted RF-based classification model was evaluated with standard classification metrics of accuracy, recall, F1 score, and AUC-ROC, as defined below:

3 $$Accuracy = \frac{TP + TN}{{TP + PN + FP + FN}},$$

4 $$Recall = \frac{TP}{{TP + FN}},$$

5 $$Precision = \frac{TP}{{TP + FP}},$$

6 $$F1 \,score = \frac{2 \,Precision \times \,recall}{{Precision + recall}}.$$

Here, *TP* and *TN* represent the total count of correctly detected positive and negative events, while *FP* and *FN* denote the total count of erroneously detected positive and negative events.

To evaluate the model's discrimination capability for the two datasets, we employed ROC analysis to derive the AUC. From the plots of the false-positive rate (FPR) on the x-axis and the true positive rate (TPR) on the y-axis, the ROC curve was obtained to calculate the AUC. TPR and FPR are given as follows:

7 $$TPR = \frac{TP}{{TP + FN}} ,$$

8 $$FPR = \frac{FP}{{FP + TN}} .$$

The confusion matrix was further visualized to assess the model performance regarding the training and testing datasets.

For the regression model, we used the metrics of accuracy and AUC-ROC to evaluate the performance. Given that the mean absolute percentage error (MAPE) is defined as an error function:

9 $${\text{MAPE}} = { }\frac{{100{\text{\% }}}}{{\text{n}}}\mathop \sum \limits_{{{\text{i}} = 1}}^{{\text{n}}} |\frac{{{\hat{\text{y}}} - {\text{y}}}}{{\text{y}}}|,$$

where y and ŷ denote the clinically measured value and the ML-predicted value of the cardiac function parameters, respectively; n is the quantity of the test dataset; thus, the accuracy is given as:

10 $${\text{Accuracy}} = 1 - {\text{MAPE}}.{ }$$

Moreover, we conducted consistency analysis between clinical measurements and ML-based predictions using the Bland‒Altman method. The Bland–Altman analysis reveals the trends, clustering patterns, and correlations of parameters between the clinical measurement datasets and ML-based prediction datasets. When the parameters fall within an acceptable range, good agreement is obtained between the two datasets, and then both methods can be used interchangeably [43].

## Data Availability

Not applicable.

## Abbreviations

LVE: Left ventricular enlargement
HF: Heart failure
ML: Machine learning
WRF: Weighted random forest
LVDdI: Left ventricular diastolic diameter index
AUC-ROC: Area under the receiver operating characteristic curve
CVD: Cardiovascular disease
CMR: Cardiac magnetic resonance
TTE: Transthoracic echocardiography
CVS: Cardiovascular system
MSE: Mean squared error
LAE: Left atrial enlargement
ASE: American Society of Echocardiography
SVM: Support vector machine model
FCNN: Fully convolutional neural network
Dense Net: Densely connected convolutional networks
FPR: False positive rate
TPR: True positive rate
MAPE: Mean absolute percentage error
